# Supplementary material for: Glycome profiling and immunohistochemistry uncover changes in cell walls of Arabidopsis thaliana roots during spaceflight
Source: NPJ Microgravity. 2023 Aug 22;9:68. doi: 10.1038/s41526-023-00312-0 (PMC10444889; doi:10.1038/s41526-023-00312-0)
Supplement: Supplementary file 1 — Reporting Summary [file 41526_2023_312_MOESM1_ESM.pdf]

# nature portfolio

Corresponding author(s): Elison Blancaflor
Last updated by author(s): Jun 27, 2023

# Reporting Summary

Nature Portfolio wishes to improve the reproducibility of the work that we publish. This form provides structure for consistency and transparency in reporting. For further information on Nature Portfolio policies, see our Editorial Policies and the Editorial Policy Checklist.

## Statistics

For all statistical analyses, confirm that the following items are present in the figure legend, table legend, main text, or Methods section.

| n/a | Confirmed | |
|---|---|---|
| ☐ | ☒ | The exact sample size (*n*) for each experimental group/condition, given as a discrete number and unit of measurement |
| ☐ | ☒ | A statement on whether measurements were taken from distinct samples or whether the same sample was measured repeatedly |
| ☐ | ☒ | The statistical test(s) used AND whether they are one- or two-sided<br>*Only common tests should be described solely by name; describe more complex techniques in the Methods section.* |
| ☒ | ☐ | A description of all covariates tested |
| ☒ | ☐ | A description of any assumptions or corrections, such as tests of normality and adjustment for multiple comparisons |
| ☐ | ☒ | A full description of the statistical parameters including central tendency (e.g. means) or other basic estimates (e.g. regression coefficient) AND variation (e.g. standard deviation) or associated estimates of uncertainty (e.g. confidence intervals) |
| ☐ | ☒ | For null hypothesis testing, the test statistic (e.g. *F*, *t*, *r*) with confidence intervals, effect sizes, degrees of freedom and *P* value noted<br>*Give P values as exact values whenever suitable.* |
| ☒ | ☐ | For Bayesian analysis, information on the choice of priors and Markov chain Monte Carlo settings |
| ☒ | ☐ | For hierarchical and complex designs, identification of the appropriate level for tests and full reporting of outcomes |
| ☒ | ☐ | Estimates of effect sizes (e.g. Cohen's *d*, Pearson's *r*), indicating how they were calculated |

*Our web collection on statistics for biologists contains articles on many of the points above.*

## Software and code

Policy information about availability of computer code

| | |
|---|---|
| Data collection | *Provide a description of all commercial, open source and custom code used to collect the data in this study, specifying the version used OR state that no software was used.* |
| Data analysis | The open source software Image J from the National Institutes of Health (NIH) was used to quantify fluorescence intensities from root sections for immunohistochemistry work. |

For manuscripts utilizing custom algorithms or software that are central to the research but not yet described in published literature, software must be made available to editors and reviewers. We strongly encourage code deposition in a community repository (e.g. GitHub). See the Nature Portfolio guidelines for submitting code & software for further information.

## Data

Policy information about availability of data

All manuscripts must include a data availability statement. This statement should provide the following information, where applicable:

- Accession codes, unique identifiers, or web links for publicly available datasets
- A description of any restrictions on data availability
- For clinical datasets or third party data, please ensure that the statement adheres to our policy

Data  generated in this study are included in this article as a supplemental table and available from the authors on reasonable request. The raw RNA-Seq data for

APEX 03-1, in which seedling debris for glycome profiling were collected, can be found at NASA GeneLab OSD-615 in the link https://osdr.nasa.gov/bio/repo/data/studies/OSD-615 with digital object identifier (DOI) OSD-615 DOI 10.26030/2746-4n42.

## Research involving human participants, their data, or biological material

Policy information about studies with human participants or human data. See also policy information about sex, gender (identity/presentation), and sexual orientation and race, ethnicity and racism.

| | |
|---|---|
| Reporting on sex and gender | *Use the terms sex (biological attribute) and gender (shaped by social and cultural circumstances) carefully in order to avoid confusing both terms. Indicate if findings apply to only one sex or gender; describe whether sex and gender were considered in study design; whether sex and/or gender was determined based on self-reporting or assigned and methods used. Provide in the source data disaggregated sex and gender data, where this information has been collected, and if consent has been obtained for sharing of individual-level data; provide overall numbers in this Reporting Summary. Please state if this information has not been collected. Report sex- and gender-based analyses where performed, justify reasons for lack of sex- and gender-based analysis.* |
| Reporting on race, ethnicity, or other socially relevant groupings | *Please specify the socially constructed or socially relevant categorization variable(s) used in your manuscript and explain why they were used. Please note that such variables should not be used as proxies for other socially constructed/relevant variables (for example, race or ethnicity should not be used as a proxy for socioeconomic status). Provide clear definitions of the relevant terms used, how they were provided (by the participants/respondents, the researchers, or third parties), and the method(s) used to classify people into the different categories (e.g. self-report, census or administrative data, social media data, etc.) Please provide details about how you controlled for confounding variables in your analyses.* |
| Population characteristics | *Describe the covariate-relevant population characteristics of the human research participants (e.g. age, genotypic information, past and current diagnosis and treatment categories). If you filled out the behavioural & social sciences study design questions and have nothing to add here, write "See above."* |
| Recruitment | *Describe how participants were recruited. Outline any potential self-selection bias or other biases that may be present and how these are likely to impact results.* |
| Ethics oversight | *Identify the organization(s) that approved the study protocol.* |

Note that full information on the approval of the study protocol must also be provided in the manuscript.

# Field-specific reporting

Please select the one below that is the best fit for your research. If you are not sure, read the appropriate sections before making your selection.

☒ Life sciences ☐ Behavioural & social sciences ☐ Ecological, evolutionary & environmental sciences

For a reference copy of the document with all sections, see nature.com/documents/nr-reporting-summary-flat.pdf

# Life sciences study design

All studies must disclose on these points even when the disclosure is negative.

| | |
|---|---|
| Sample size | No sample size calculation was performed for the glycome profiling component of the study. Sample size (i.e., number of seedlings) was determined based on the total growing area of the 90 mm x 90 mm square Petri dish to allow best possible growth within the 6 and 11 day growth period in the Veggie hardware. Each Petri dish had 18 seedlings and each treatment (spaceflight versus ground controls) had three Petri dishes. The ground procedural controls described in the manuscript and mentioned under "replication" below showed that this sample size was sufficient. |
| Data exclusions | No data were excluded from the analyses. |
| Replication | This was a one time spaceflight experiment, and there was no opportunity for technical replication. However, the experiment was designed in the plant spaceflight hardware (Veggie) that allowed three biological replicates for the glycome profiling experiments, while immunohistochemistry data was obtained from three root sections for each glycan antibody and treatment (i.e., ground versus spaceflight). As noted in detail in the manuscript, measures to verify reproducibility for glycome profiling came from two procedural ground controls to increase confidence that plant debris from RNA columns faithfully reported one time experiments in space. For immunohistochemistry a fixation control was used as procedural ground control. |
| Randomization | Randomization was not relevant to the study because there were only two major treatments, namely, spaceflight versus ground controls. Within each treatment, Five petri dishes with seedlings were positioned on vertical carriers on the left and right side of the Veggie hardware on the International Space Station and on the ground. |
| Blinding | Blinding was not possible because sample tubes for collecting seedlings had to be clearly labeled preflight for the International Space Station (ISS) crew to harvest seedlings correctly. Pre-labeled collection tubes were then returned to the investigator team for processing. |

# Reporting for specific materials, systems and methods

We require information from authors about some types of materials, experimental systems and methods used in many studies. Here, indicate whether each material, system or method listed is relevant to your study. If you are not sure if a list item applies to your research, read the appropriate section before selecting a response.

## Materials & experimental systems

| n/a | Involved in the study |
|---|---|
| ☐ | ☒ Antibodies |
| ☒ | ☐ Eukaryotic cell lines |
| ☒ | ☐ Palaeontology and archaeology |
| ☒ | ☐ Animals and other organisms |
| ☒ | ☐ Clinical data |
| ☒ | ☐ Dual use research of concern |
| ☐ | ☒ Plants |

## Methods

| n/a | Involved in the study |
|---|---|
| ☒ | ☐ ChIP-seq |
| ☒ | ☐ Flow cytometry |
| ☒ | ☐ MRI-based neuroimaging |

## Antibodies

Antibodies used

Monoclonal antibodies used came from the Complex Carbohydrate Research Center (CCRC) at the University of Georgia. The generation of these cell wall antibodies are described in more detail in previous papers of the co-authors Pattathil, Avci, and Hahn that were cited in the paper. There were more than 150 antibodies used for glycome profiling and 22 for the immunohistochemistry. Clone name of each antibody and respective epitope are noted in the main and supplementary figures of the manuscript.

Validation

See papers of co-authors Pattathil, Avci, and Hahn that were cited in the paper for validation information. The epitopoe for all antibodies used have already been validated by numerous publications some of which are cited in the current manuscript.

## Dual use research of concern

Policy information about dual use research of concern

### Hazards

Could the accidental, deliberate or reckless misuse of agents or technologies generated in the work, or the application of information presented in the manuscript, pose a threat to:

| No | Yes |
|---|---|
| ☒ | ☐ Public health |
| ☒ | ☐ National security |
| ☒ | ☐ Crops and/or livestock |
| ☒ | ☐ Ecosystems |
| ☒ | ☐ Any other significant area |

### Experiments of concern

Does the work involve any of these experiments of concern:

| No | Yes |
|---|---|
| ☒ | ☐ Demonstrate how to render a vaccine ineffective |
| ☒ | ☐ Confer resistance to therapeutically useful antibiotics or antiviral agents |
| ☒ | ☐ Enhance the virulence of a pathogen or render a nonpathogen virulent |
| ☒ | ☐ Increase transmissibility of a pathogen |
| ☒ | ☐ Alter the host range of a pathogen |
| ☒ | ☐ Enable evasion of diagnostic/detection modalities |
| ☒ | ☐ Enable the weaponization of a biological agent or toxin |
| ☒ | ☐ Any other potentially harmful combination of experiments and agents |
