# Supplementary material for: Glycome profiling and immunohistochemistry uncover changes in cell walls of Arabidopsis thaliana roots during spaceflight
Source: NPJ Microgravity. 2023 Aug 22;9:68. doi: 10.1038/s41526-023-00312-0 (PMC10444889; doi:10.1038/s41526-023-00312-0)
Supplement: Supplementary file 2 — Supplementary Information [file 41526_2023_312_MOESM2_ESM.pdf]

Supplemental Information-Nakashima et al

Glycome profiling and immunohistochemistry uncover spaceflight-induced changes in non-cellulosic cell wall components in *Arabidopsis thaliana* seedling roots

Jin Nakashima[1], Sivakumar Pattathil[2, 3], Utku Avci[3, 4], Sabrina Chin[5], J. Alan Sparks[6], Michael G. Hahn[4], Simon Gilroy[5] and Elison B. Blancaflor[7]

[1]Analytical Instrumentation Facility, North Carolina State University, 2410 Camp, US Shore Drive, Raleigh, NC 27606 USA

[2]Mascoma LLC (Lallemand Inc.), 67 Etna Road, Lebanon, NH 03766, USA

[3]The University of Georgia, Complex Carbohydrate Research Center, 315 Riverbend Rd, Athens, GA, 30602, USA

[4]Department of Agricultural Biotechnology, Faculty of Agriculture, Eskisehir Osmangazi University, Eskisehir 26160, TURKEY

[5]Department of Botany, 430 Lincoln Drive, University of Wisconsin, Madison, WI 53706, USA

[6]Noble Research Institute LLC, 2510 Sam Noble Parkway, Ardmore, OK 73401, USA

[7]Utilization & Life Sciences Office, Exploration Research and Technology Programs, NASA John F. Kennedy Space Center, FL, 32899, USA

Running title: Spaceflight-induced plant cell wall changes

Correspondence to: Elison B. Blancaflor,Utilization & Life Sciences Office,

NASA John F. Kennedy Space Center,

Merritt Island, Florida, USA

Tel no. (321)-867-4847

e-mail: elison.b.blancaflor@nasa.gov

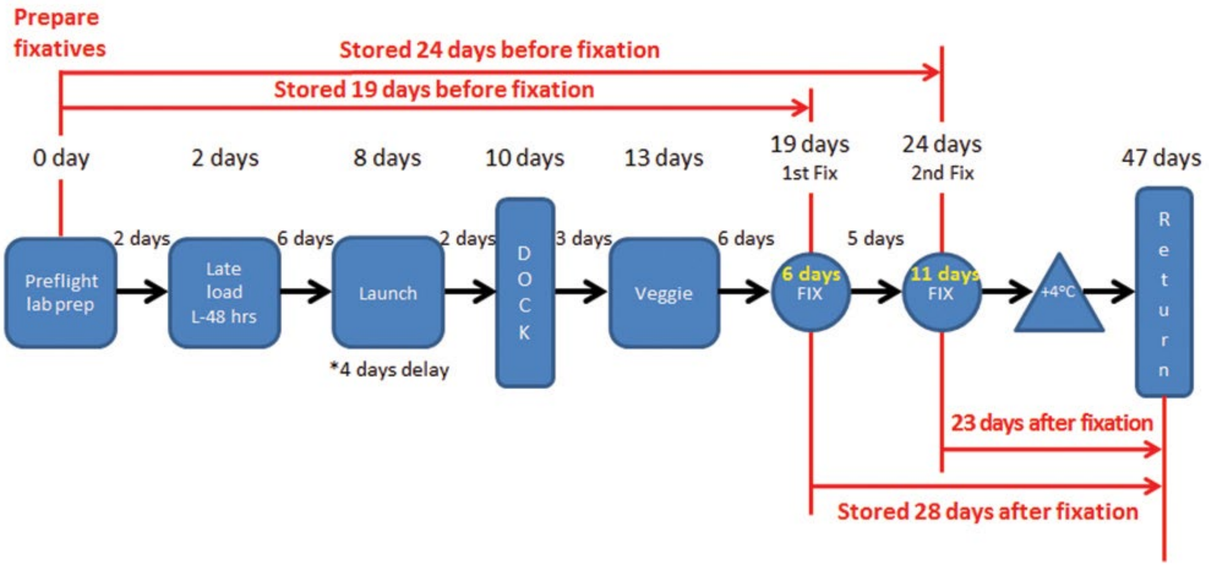

Supplementary Fig. 1 Schematic diagram of the experimental timeline of Advanced Plant EXperiments (APEX) 03-1. Note that the RNA*later* and aldehydes were stored for 19 and 24 days in Kennedy Fixation Tubes (KFTs) prior to seedling fixation on the International Space Station. The experiment was activated by transferring square Petri dishes from 4 °C and darkness to the Veggie unit under continuous white light and 23 °C. Exposure to white light and higher temperatures triggered seeds to germinate on orbit and the ground. Petri dishes were kept in a vertical orientation in Veggie. Fixation was done at 6 and 11 days after experiment activation by transferring seedlings to KFTs with 4% paraformaldehyde and 2.5% glutaraldehyde or RNA*later*. KFTs with aldehydes and RNA*later* were stored at 4 °C and -80 °C, respectively. Seedlings fixed at 6 and 11 days were returned at 28 and 23 days, respectively, for processing.

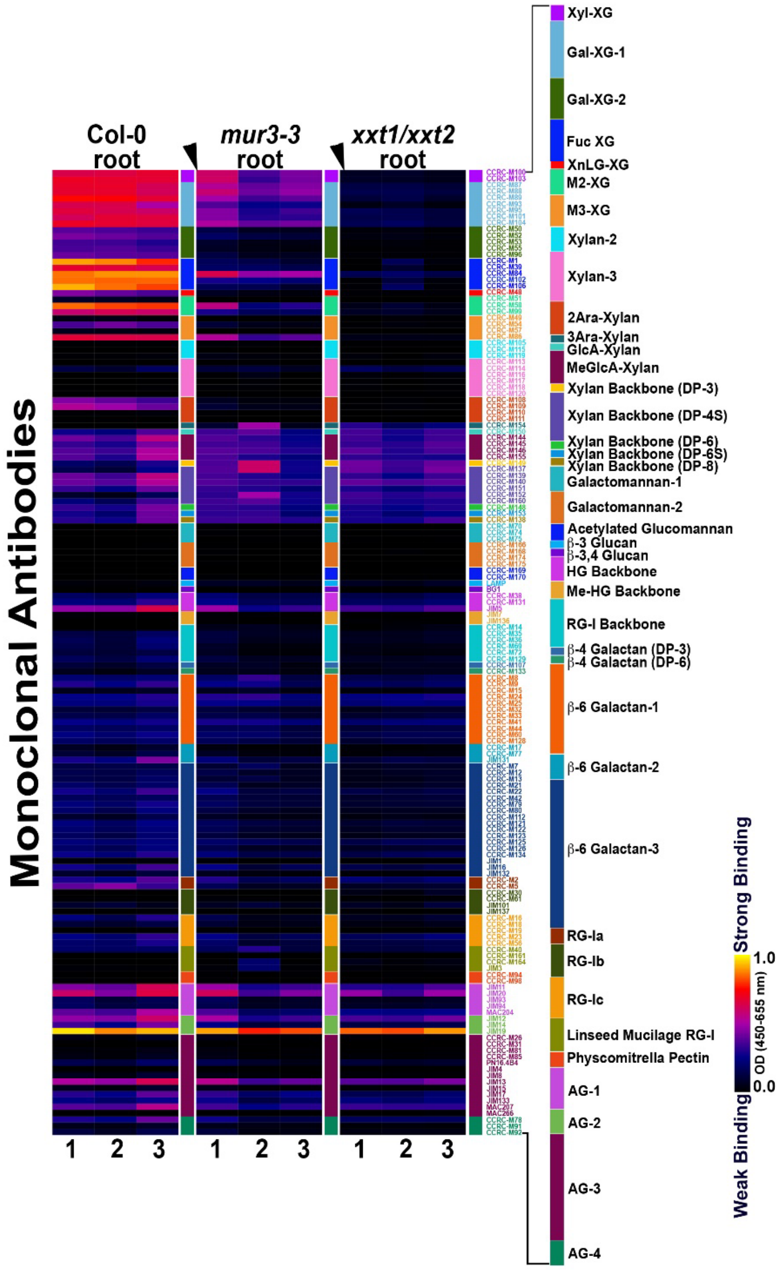

Supplementary Fig. 2 Glycome profiling of root cell wall extracts of two xyloglucan mutants from QIAshredder spin column and processed with the single 4M KOH step. Cell wall extracts from the *xxt1/xxt2* double mutant, which makes no xyloglucan, and *mur3-3* single mutant, which lacks galactose-fucose xyloglucan side chains, have lower binding to xyloglucan mAbs (arrowheads) than extracts of wild type seedlings.

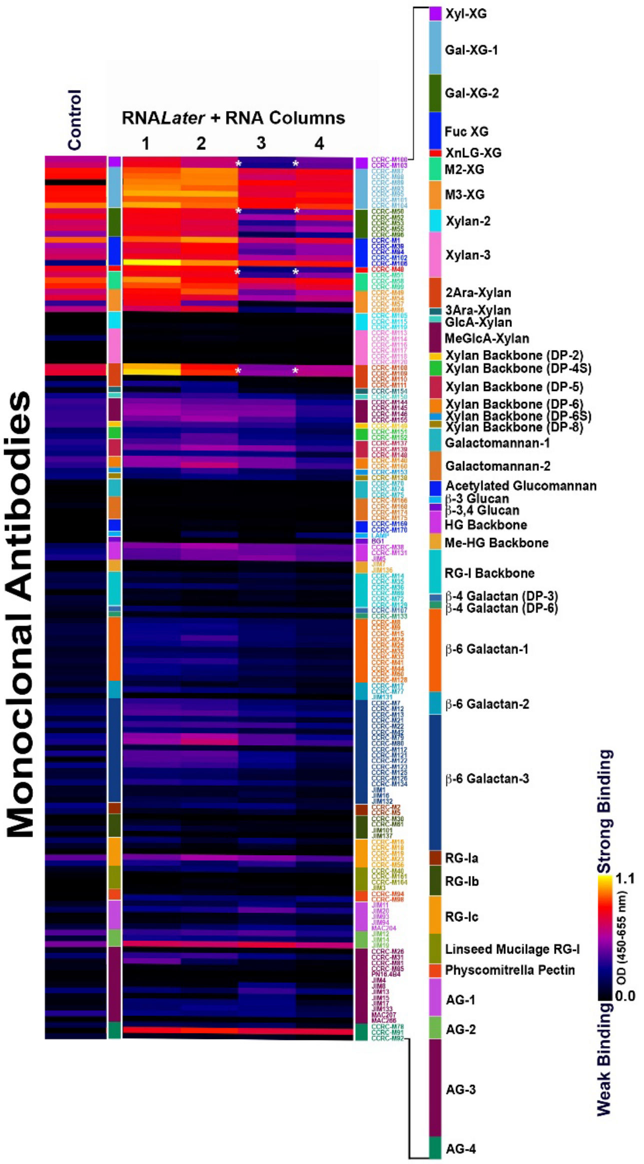

Supplementary Fig. 3 Glycome profiling heatmaps of whole seedlings directly processed with the single 4M KOH step (controls) and RNA*Later*-fixed whole seedling extracts from RNA columns and processed with 4M KOH (lanes 1-4). Note the depletion of some xylan and xyloglucans in samples from lanes 3 and 4 that were fixed in RNA*Later* and whole seedling debris obtained from RNA columns (asterisks).

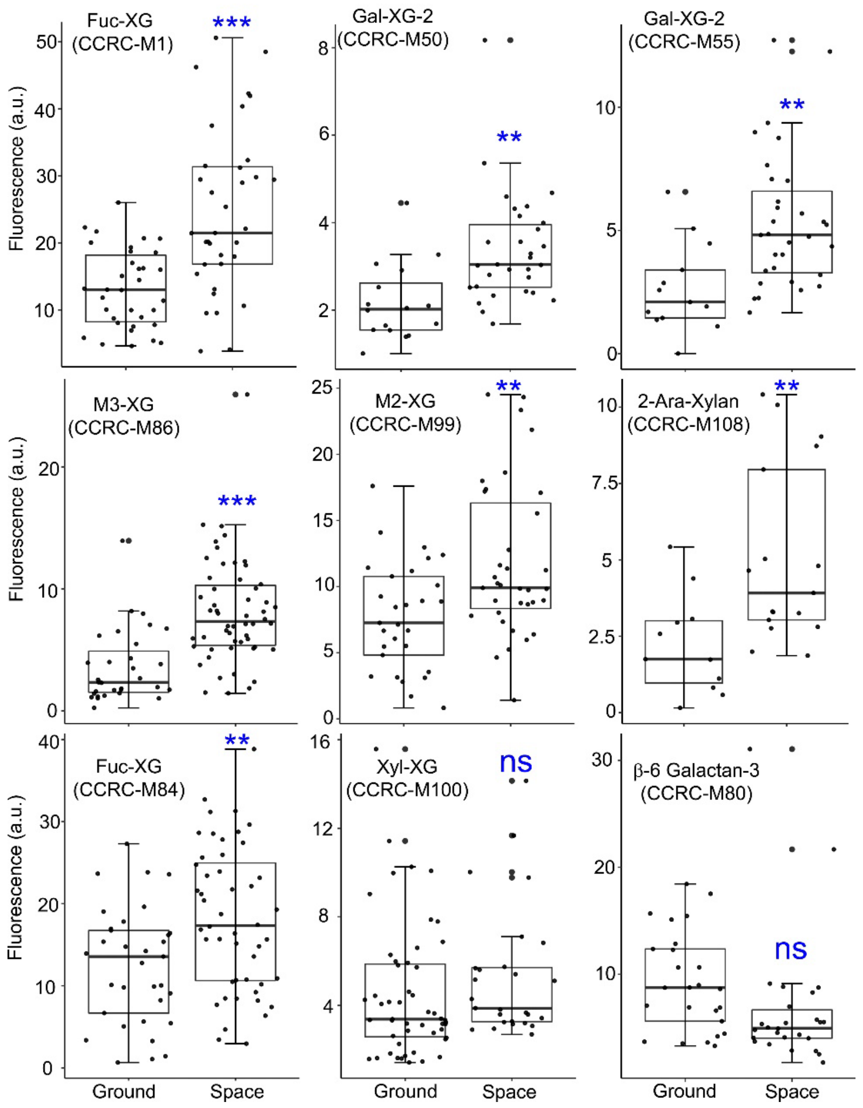

Supplementary Fig. 4 Quantification of root tip longitudinal section fluorescence from space- and Earth-grown seedlings labeled with mAbs to non-cellulosic glycans. Box limits indicate 25th and 75th percentiles, horizontal line is the median, and whiskers display minimum and maximum values. ***P<0.0001, **P <0.001, and *P<0.01 indicate statistical significance as determined by Student's t-test. Not significant (ns). Each dot represents individual measurement from 30-50 regions of three root tip longitudinal sections. Xyloglucan (XG); Galactose (Gal); Fucose (Fuc); Arabinose (Ara); Xylose (Xyl).

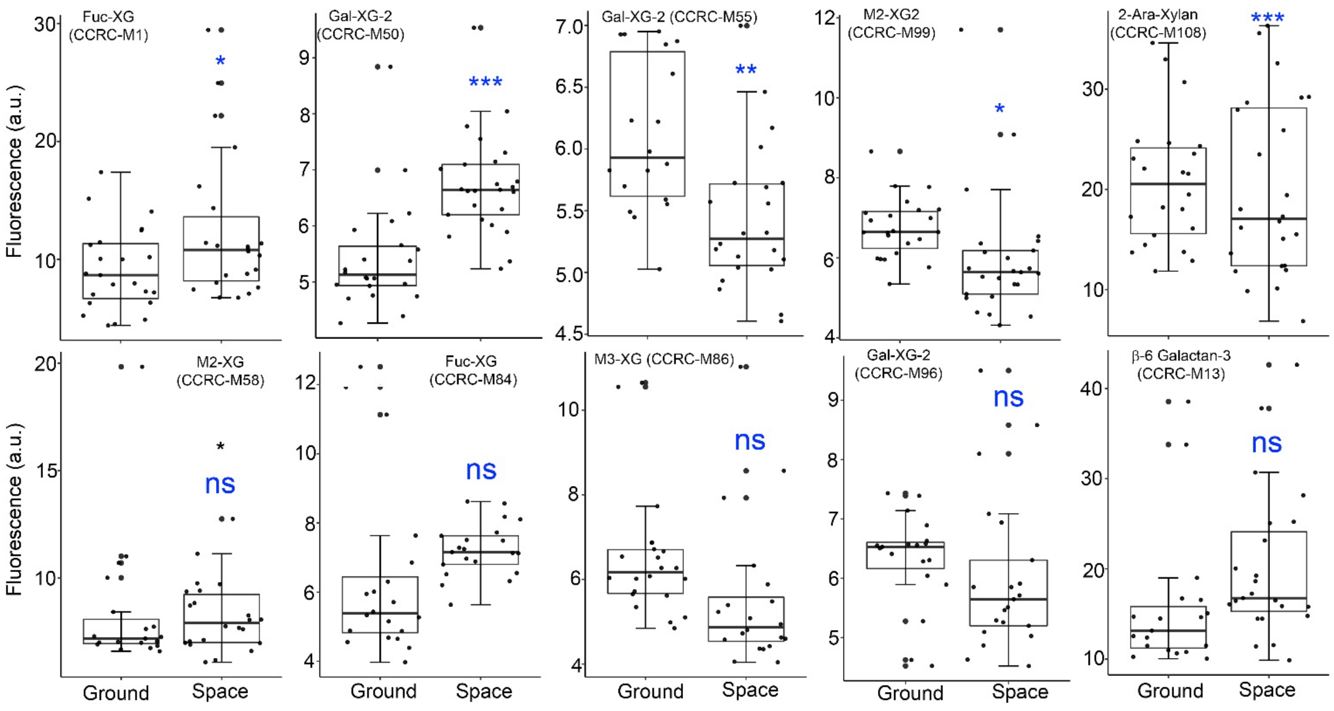

Supplementary Fig. 5 Quantification of root cross section fluorescence from space- and Earth-grown seedlings labeled with mAbs to non-cellulosic glycans. Box limits indicate 25th and 75th percentiles, horizontal line is the median, and whiskers display minimum and maximum values. ***P<0.0001, **P <0.001, and *P<0.01 indicate statistical significance as determined by Student's t test. Not significant (ns). Each dot represents individual measurement from 20-30 regions of three root cross sections. Xyloglucan (XG); Galactose (Gal); Fucose (Fuc); Arabinose (Ara); Xylose (Xyl).

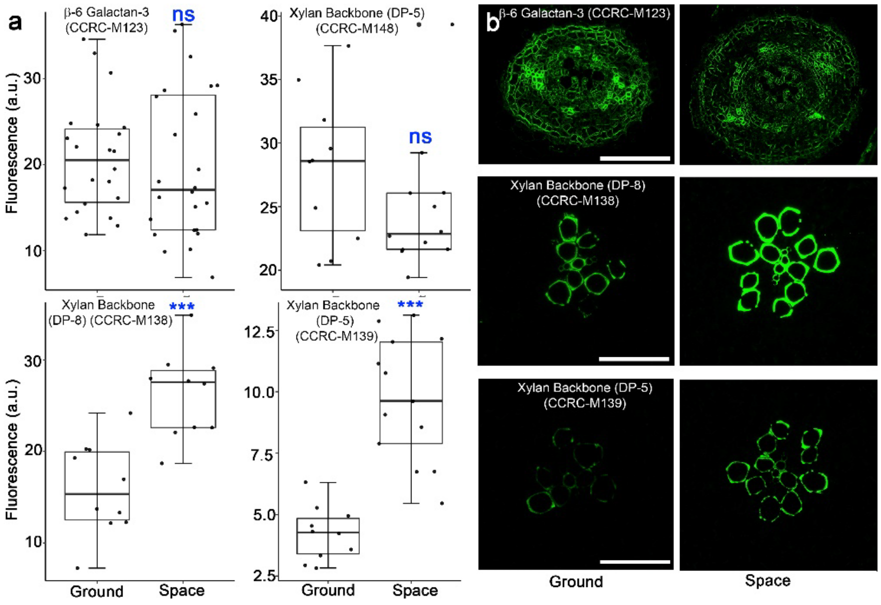

Supplementary Fig. 6 Immunohistochemistry of root cross sections from space- and Earth-grown seedlings labeled with mAbs to non-cellulosic glycans. (a) Fluorescence of root cross sections labeled with CCRC-M123 and CCRC-M148 were not significantly different statistically between space and ground controls. In some cases, different root sections taken from the same root tissue block were labeled independently with different mAbs (e.g., CCRC-M123 shown here and CCRC-M79 in Fig. 6a). Root cross sections from seedlings grown in space labeled with CCRC-M138 and CCRC-M139 had higher fluorescence than those of the ground controls. Box limits indicate 25th and 75th percentiles, horizontal line is the median, and whiskers display minimum and maximum values. ***P<0.0001 indicate statistical significance as determined by Student's t test. Not significant (ns). Each dot represents individual measurement from 20-30 regions of three root cross sections. (b) CCRC-M123 labels roots cells uniformly in space and on Earth. The xylan mAbs, CCRC-M138 and 139, preferentially labels root xylem cells in space and on Earth (arrows). Note that xylem cells of roots from space-grown seedlings labeled with CCRC-M138 and 139 are more intensely labeled than that of the ground controls. Bar in top panel of b = 50 μm; Bars in middle and bottom panel of b = 20 μm. . Degree of Polymerization (DP).

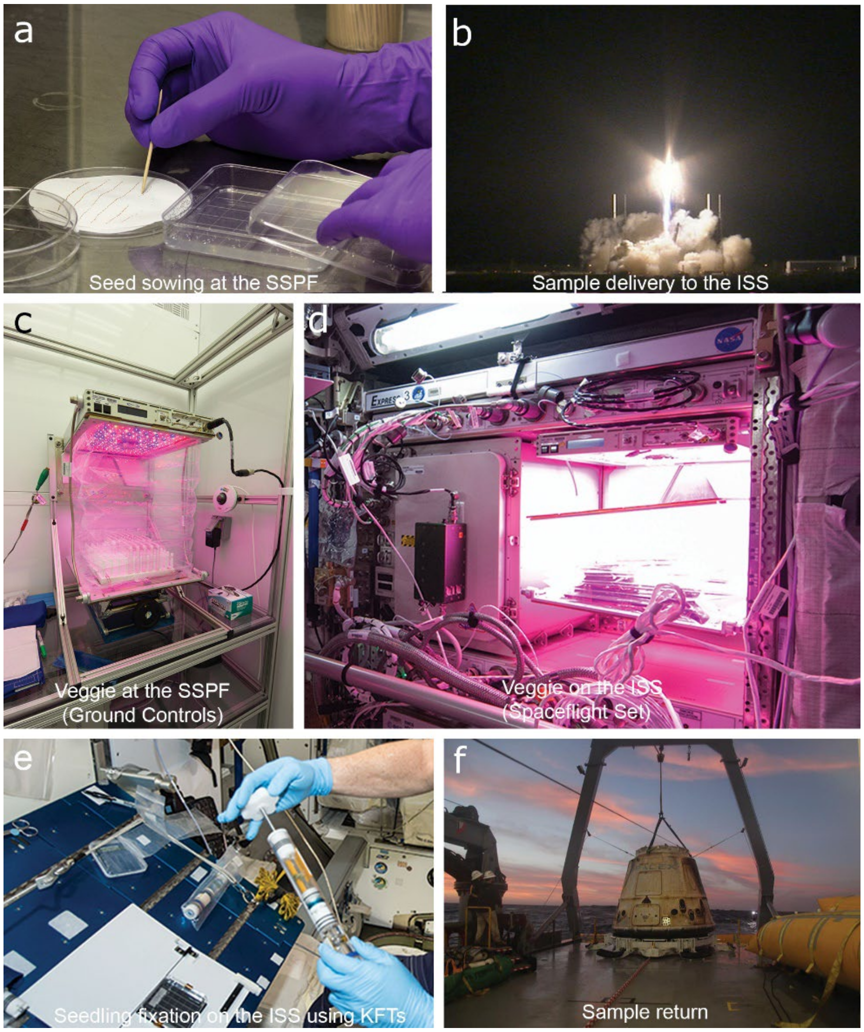

Supplementary Fig. 7 Overview of preflight, flight and postflight operations for glycome profiling and immunohistochemistry of *A. thaliana* seedling roots. (a) Planting of *A. thaliana* seeds in square Petri dishes under a laminar flow hood at the Space Station Processing Facility (SSPF) eight days prior to Space X-5 launch. (b) Launch of the SpaceX Falcon rocket carrying the Dragon spacecraft with the Petri dishes containing *A. thaliana* seeds. (c) Veggie hardware at the SPFF used for the ground controls. (d) Veggie hardware located at the Columbus module of the International Space Station (ISS). (e) Harvesting 11-day-old seedlings and transferring them to Kennedy Fixation Tubes (KFTs) containing chemical fixatives. (f) The Dragon spacecraft with the APEX 03-1 *A. thaliana* seedlings being lifted onto the deck of a recovery ship in the Pacific Ocean. Images in panels b, d, e, and f are courtesy of NASA and are in the public domain.

Supplementary Table 1 - Raw ELISA readout of monoclonal antibodies (mAbs) used to generate the heatmaps shown in Fig. 2a and Fig. 2b. Each column representing one biological replicate is an average of two technical replicates. The average of three biological replicates in this table were used to plot the bar graphs shown in Fig. 2c.

	GROUND						SPACE					
**mAbs**	**R7(6d)**	**R11(6d)**	**R10(6d)**	**R9(11d)**	**R2(11d)**	**R4(11d)**	**R6(6d)**	**R1(6d)**	**R3(6d)**	**R5(11d)**	**R8(11d)**	**R12(11d)**
CCRC-M95	0.7925	0.9955	0.782	0.8925	0.9315	0.8185	0.7805	0.999	0.8005	0.907	0.908	1.015
CCRC-M101	0.7865	0.943	0.7565	0.8965	0.9275	0.828	0.784	0.933	0.7285	0.9295	0.898	0.982
CCRC-M104	0.8855	1.038	0.796	0.9615	1.019	0.955	0.8805	0.997	0.7795	1.036	0.8945	1.0515
CCRC-M89	0.8245	0.847	0.7415	0.905	0.8995	0.8575	0.743	0.848	0.7115	0.9975	0.8615	0.882
CCRC-M93	0.747	0.8165	0.667	0.861	0.871	0.801	0.693	0.8645	0.653	0.9295	0.8755	0.844
CCRC-M87	0.7875	0.862	0.7665	0.901	0.8975	0.915	0.827	0.8715	0.763	0.9755	0.9355	0.8885
CCRC-M88	0.7905	0.8405	0.7075	0.9155	0.9075	0.8725	0.7665	0.872	0.765	0.9895	0.907	0.869
CCRC-M100	0.5715	0.7655	0.451	0.7505	0.7685	0.571	0.5505	0.7525	0.481	0.8255	0.755	0.8415
CCRC-M103	0.5505	0.713	0.4345	0.73	0.692	0.5845	0.5285	0.6805	0.4405	0.7995	0.7565	0.7395
CCRC-M58	0.816	0.997	0.7375	0.933	0.963	0.921	0.86	0.915	0.7445	0.999	0.9195	1.009
CCRC-M86	0.456	0.5375	0.3615	0.656	0.568	0.4955	0.444	0.5825	0.3705	0.734	0.6695	0.6205
CCRC-M55	0.53	0.7375	0.3855	0.749	0.6965	0.5955	0.507	0.7155	0.3605	0.7765	0.744	0.808
CCRC-M52	0.5995	0.8025	0.498	0.765	0.791	0.6565	0.5705	0.773	0.483	0.7785	0.764	0.8075
CCRC-M99	0.7575	0.902	0.7055	0.872	0.9615	0.756	0.711	0.9285	0.677	0.833	0.8655	0.9275
CCRC-M54	0.6235	0.8595	0.483	0.7805	0.8205	0.643	0.5845	0.8075	0.47	0.83	0.7715	0.8895
CCRC-M48	0.4325	0.6715	0.3465	0.688	0.6465	0.5035	0.4355	0.6765	0.3445	0.7375	0.6985	0.7905
CCRC-M49	0.107	0.211	0.057	0.179	0.173	0.136	0.0905	0.194	0.0625	0.1995	0.172	0.198
CCRC-M96	0.2805	0.509	0.1705	0.6165	0.494	0.305	0.217	0.494	0.1745	0.594	0.59	0.6065
CCRC-M50	0.571	0.7665	0.473	0.7755	0.799	0.624	0.551	0.765	0.466	0.719	0.7485	0.7735
CCRC-M51	0.021	0.0795	0.0065	0.16	0.046	0.0245	0.0115	0.0615	0.0105	0.226	0.163	0.16
CCRC-M53	0.51	0.685	0.3865	0.679	0.6855	0.5125	0.4525	0.675	0.3765	0.7	0.6805	0.7355
CCRC-M57	0.0165	0.067	0.004	0.124	0.0355	0.023	0.0125	0.05	0.0125	0.2045	0.119	0.113
CCRC-M102	0.7115	0.878	0.5625	0.8355	0.8215	0.737	0.6705	0.7625	0.636	0.926	0.8335	0.927
CCRC-M39	0.4495	0.5745	0.4575	0.5915	0.5835	0.535	0.433	0.5425	0.4305	0.656	0.5985	0.5975
CCRC-M106	0.9075	0.9885	0.7995	0.9675	1.003	0.881	0.8845	1.0095	0.727	1.0415	0.9175	1.0305
CCRC-M84	0.8205	0.954	0.8015	1.0265	1.0055	0.935	0.817	1.0355	0.808	1.037	1.003	1.007
CCRC-M1	0.854	0.9865	0.7855	0.969	1.123	0.924	0.853	1.1035	0.9075	0.97	0.965	1.0505
CCRC-M111	0	0.0025	0	0.0035	0.00225	0	0.00125	0.00525	0	0.00325	0.00325	0.009
CCRC-M108	0.523	0.718	0.277	0.7385	0.6675	0.499	0.411	0.7295	0.2775	0.7685	0.774	0.778
CCRC-M109	0.6015	0.7755	0.425	0.7825	0.743	0.603	0.5415	0.796	0.386	0.84	0.801	0.8175

CCRC-M119	0.0015	0.0125	0.003	0.009	0.006	0.0035	0.003	0.006	0.003	0.0045	0.0095	0.0045
CCRC-M115	0.024	0.0245	0.0105	0.034	0.0185	0.019	0.0195	0.0255	0.0165	0.0295	0.026	0.0175
CCRC-M110	0.0085	0.008	0	0.0145	0.021	0.004	0.01	0.0125	0.0125	0.017	0.004	0.008
CCRC-M105	0.025	0.039	0.025	0.0235	0.0335	0.026	0.0325	0.027	0.0305	0.032	0.0275	0.019
CCRC-M117	0	0.001	0.0005	0	0.004	0	0	0.0025	0	0	0	0
CCRC-M113	0.007	0.025	0.007	0.0155	0.0135	0.0245	0.015	0.0065	0.012	0.0165	0.021	0.013
CCRC-M120	0.0125	0.016	0.012	0.026	0.0155	0.0135	0.012	0.0085	0.032	0.022	0.018	0.019
CCRC-M118	0.0065	0.0085	0.0065	0.0555	0.0325	0.0255	0.0065	0.015	0.01	0.05	0.0385	0.0355
CCRC-M116	0.008	0.0105	0.006	0.005	0.014	0.0115	0.0075	0.0155	0.011	0.005	0.0045	0.0075
CCRC-M114	0.1545	0.2565	0.0875	0.384	0.2435	0.208	0.096	0.272	0.0935	0.4005	0.374	0.313
CCRC-M154	0.2235	0.2685	0.2065	0.242	0.3235	0.266	0.2305	0.3305	0.206	0.233	0.2255	0.2555
CCRC-M150	0.2535	0.277	0.172	0.3595	0.2855	0.276	0.1585	0.26	0.1985	0.418	0.349	0.35
CCRC-M144	0.331	0.4665	0.238	0.4675	0.354	0.342	0.2795	0.287	0.2605	0.478	0.518	0.4455
CCRC-M146	0.4055	0.4895	0.318	0.525	0.3635	0.375	0.3625	0.344	0.312	0.528	0.4885	0.47
CCRC-M145	0.4325	0.5275	0.3565	0.51	0.427	0.433	0.389	0.371	0.3525	0.507	0.481	0.462
CCRC-M155	0.301	0.31	0.2715	0.3815	0.405	0.304	0.27	0.3705	0.2445	0.395	0.4	0.3715
CCRC-M153	0.19	0.1965	0.1325	0.2765	0.238	0.188	0.1505	0.204	0.1345	0.3505	0.309	0.331
CCRC-M151	0.189	0.2125	0.124	0.328	0.165	0.1865	0.1465	0.155	0.118	0.4055	0.322	0.32
CCRC-M148	0.27	0.338	0.2245	0.3945	0.308	0.275	0.2235	0.245	0.181	0.4725	0.4085	0.4475
CCRC-M140	0.402	0.496	0.289	0.579	0.409	0.4345	0.2875	0.335	0.305	0.6905	0.5625	0.538
CCRC-M139	0.342	0.4445	0.2175	0.509	0.3215	0.34	0.2695	0.274	0.2345	0.592	0.529	0.435
CCRC-M138	0.25	0.336	0.181	0.3175	0.2845	0.2565	0.2105	0.2455	0.186	0.4695	0.384	0.414
CCRC-M160	0.439	0.4025	0.4105	0.4785	0.4415	0.402	0.3805	0.477	0.349	0.471	0.4815	0.416
CCRC-M137	0.296	0.3685	0.255	0.412	0.2685	0.274	0.265	0.232	0.2325	0.4695	0.408	0.408
CCRC-M152	0.2925	0.3065	0.2645	0.3385	0.384	0.286	0.2645	0.332	0.25	0.364	0.309	0.3775
CCRC-M149	0.2395	0.293	0.182	0.36	0.2585	0.26	0.2125	0.23	0.18	0.407	0.3635	0.364
CCRC-M75	0	0.00675	0.00775	0.00025	0.0065	0.00325	0	0.006	0.00375	0.0095	0.00175	0.00575
CCRC-M70	0.004	0.004	0.002	0	0.00275	0.001	0	0.00075	0.013	0.0055	0.001	0
CCRC-M74	0.003	0.0105	0.006	0.0035	0	0	0.003	0.001	0	0	0.012	0.001
CCRC-M166	0.003	0.0005	0.02925	0.00025	0	0.00125	0	0	0.00025	0	0.00075	0.00025
CCRC-M168	0.022	0.0115	0.012	0.007	0.0085	0.0095	0.0095	0.0075	0.0165	0.009	0.0065	0.0085
CCRC-M174	0.0015	0	0.001	0	0.003	0	0.002	0.0005	0.00075	0.001	0.00025	0
CCRC-M175	0	0.00325	0.02775	0	0.002	0	0	0	0.008	0.0065	0.00225	0.00125
CCRC-M169	0.0135	0.022	0.016	0.0165	0.0095	0.02	0.0185	0.0125	0.016	0.0155	0.0125	0.0205

CCRC-M170	0.0025	0.0075	0.059	0.002	0.002	0.0015	0.002	0.00025	0.017	0.0015	0.00025	0.004
LAMP	0	0	0	0	0	0.001	0	0.002	0.004	0	0.0005	0
BG1	0.056	0.044	0.009	0.0225	0.0995	0.0905	0.021	0.254	0.046	0.031	0.036	0.221
CCRC-M131	0.285	0.445	0.2275	0.446	0.3965	0.323	0.2265	0.342	0.1895	0.411	0.41	0.451
CCRC-M38	0.2765	0.367	0.238	0.332	0.3375	0.2955	0.2475	0.2985	0.202	0.366	0.327	0.3365
JIM5	0.099	0.1955	0.0615	0.1925	0.1285	0.11	0.023	0.1275	0.0605	0.1705	0.1775	0.189
JIM136	0.0035	0.0055	0.0025	0.0035	0.001	0.0025	0.002	0.0025	0.0045	0.003	0.003	0.003
JIM7	0.0025	0.0055	0.003	0.0025	0	0.0075	0.0035	0.0005	0.0065	0.0065	0.0005	0.0055
CCRC-M69	0.063	0.084	0.0345	0.1245	0.083	0.054	0.042	0.0795	0.025	0.134	0.1025	0.118
CCRC-M35	0.1095	0.1315	0.0765	0.177	0.115	0.0975	0.067	0.116	0.071	0.17	0.137	0.1435
CCRC-M36	0.105	0.139	0.061	0.171	0.115	0.0935	0.056	0.118	0.047	0.176	0.155	0.148
CCRC-M14	0.0105	0.06	0.0115	0	0	0.0345	0	0	0.051	0	0	0.0295
CCRC-M129	0.109	0.167	0.0625	0.1685	0.1285	0.103	0.067	0.1225	0.0525	0.193	0.1515	0.1875
CCRC-M72	0.0515	0.078	0.0205	0.1125	0.0605	0.045	0.0295	0.053	0.0155	0.1155	0.085	0.093
JIM3	0.005	0.0125	0.0035	0.013	0.0395	0.012	0.0075	0.025	0.008	0.041	0.019	0.019
CCRC-M40	0.1135	0.1435	0.083	0.13	0.12	0.1415	0.0875	0.079	0.081	0.1845	0.135	0.1365
CCRC-M161	0.0115	0.014	0.007	0.0325	0.013	0.013	0.007	0.008	0.007	0.0445	0.025	0.0255
CCRC-M164	0.002	0.0025	0.0015	0.0005	0.002	0.0035	0.001	0.006	0.01	0	0.0015	0.001
CCRC-M98	0.006	0.0065	0.0045	0.005	0.001	0.0055	0.005	0.004	0.018	0.0085	0.0055	0.003
CCRC-M94	0.0063	0.0225	0.008	0.01	0.0145	0.009	0.0095	0.006	0.0145	0.016	0.009	0.0185
CCRC-M5	0.0435	0.064	0.021	0.102	0.0635	0.049	0.0275	0.0525	0.05	0.1485	0.1095	0.087
CCRC-M2	0.1705	0.2175	0.109	0.283	0.216	0.1855	0.145	0.211	0.126	0.31	0.304	0.3115
JIM137	0.0045	0.009	0.007	0.0175	0.0055	0.00475	0.0105	0.012	0.00575	0.009	0.021	0.0065
JIM101	0.114	0.145	0.076	0.078	0.066	0.134	0.0965	0.055	0.1315	0.053	0.0695	0.1015
CCRC-M61	0.0825	0.084	0.0465	0.042	0.0475	0.098	0.071	0.035	0.0985	0.038	0.0305	0.0555
CCRC-M30	0	0.011	0.055	0.01075	0.024	0.01125	0	0.0125	0.03475	0	0.00675	0.01475
CCRC-M23	0.1305	0.2625	0.1165	0.295	0.216	0.1525	0.097	0.221	0.0875	0.252	0.272	0.317
CCRC-M17	0.0008	0.01	0.0005	0.022	0.0135	0.0025	0	0.0095	0	0.05	0.0145	0.0195
CCRC-M19	0	0.00025	0	0	0.009	0.00075	0	0.0035	0	0	0.00075	0.00025
CCRC-M18	0.0145	0.0315	0.0115	0.0485	0.033	0.014	0.0045	0.036	0.012	0.097	0.04	0.0605
CCRC-M56	0.1425	0.325	0.1195	0.371	0.1695	0.13	0.1125	0.2955	0.0805	0.3845	0.3265	0.325
CCRC-M16	0.0565	0.161	0.04	0.298	0.1505	0.0695	0.029	0.1415	0.0315	0.3445	0.2285	0.2285
CCRC-M60	0.193	0.277	0.116	0.332	0.228	0.1895	0.162	0.215	0.1295	0.362	0.3095	0.2915
CCRC-M41	0.067	0.0835	0.0465	0.088	0.064	0.0725	0.0655	0.057	0.042	0.0775	0.077	0.081

CCRC-M80	0.3615	0.431	0.279	0.492	0.408	0.3625	0.299	0.4265	0.2575	0.455	0.464	0.451
CCRC-M79	0.34	0.418	0.2565	0.449	0.3945	0.337	0.272	0.394	0.251	0.436	0.446	0.4225
CCRC-M44	0.124	0.1475	0.067	0.1935	0.1365	0.1225	0.0575	0.1325	0.0605	0.2035	0.201	0.1765
CCRC-M33	0.1245	0.1615	0.082	0.2035	0.1415	0.1285	0.086	0.137	0.07	0.1865	0.1945	0.184
CCRC-M32	0.0945	0.122	0.0585	0.138	0.114	0.0925	0.062	0.11	0.0585	0.1365	0.151	0.1285
CCRC-M13	0.2945	0.4105	0.1655	0.4245	0.346	0.2975	0.228	0.426	0.185	0.4475	0.4185	0.4015
CCRC-M42	0.131	0.147	0.075	0.186	0.1305	0.116	0.078	0.1305	0.076	0.188	0.1875	0.1685
CCRC-M24	0.149	0.1935	0.108	0.252	0.1565	0.1525	0.1115	0.153	0.101	0.223	0.2345	0.246
CCRC-M12	0.3055	0.4195	0.193	0.3515	0.338	0.2485	0.2195	0.37	0.1915	0.4005	0.3675	0.3475
CCRC-M7	0.181	0.2455	0.0975	0.2595	0.1925	0.1595	0.108	0.198	0.097	0.2635	0.272	0.2495
CCRC-M77	0.1455	0.168	0.08	0.2055	0.1265	0.0895	0.072	0.141	0.072	0.2235	0.215	0.153
CCRC-M25	0.157	0.2105	0.126	0.258	0.203	0.169	0.122	0.1965	0.1225	0.223	0.2545	0.235
CCRC-M9	0.1865	0.237	0.106	0.324	0.213	0.204	0.154	0.1915	0.1295	0.349	0.318	0.276
CCRC-M128	0.165	0.253	0.0955	0.263	0.1995	0.184	0.127	0.1835	0.1125	0.305	0.266	0.284
CCRC-M126	0.1705	0.191	0.102	0.274	0.1925	0.1715	0.1265	0.179	0.0935	0.279	0.2985	0.2465
CCRC-M134	0.2395	0.331	0.149	0.38	0.2875	0.2275	0.1685	0.266	0.1475	0.4095	0.364	0.3575
CCRC-M125	0.173	0.1985	0.111	0.25	0.196	0.1695	0.132	0.1915	0.107	0.2595	0.2665	0.2275
CCRC-M123	0.337	0.3625	0.2135	0.4325	0.3635	0.3235	0.2885	0.363	0.1955	0.456	0.425	0.3845
CCRC-M122	0.42	0.409	0.274	0.4655	0.42	0.3675	0.347	0.4255	0.268	0.526	0.459	0.42
CCRC-M121	0.3485	0.387	0.2645	0.4845	0.366	0.3625	0.4145	0.3955	0.2505	0.5465	0.4465	0.4045
CCRC-M112	0.11	0.147	0.0995	0.178	0.155	0.1215	0.093	0.157	0.0945	0.167	0.174	0.15
CCRC-M21	0.1765	0.226	0.0835	0.263	0.2385	0.1755	0.1215	0.2135	0.13	0.2995	0.287	0.2415
JIM131	0.1005	0.128	0.0615	0.169	0.117	0.0865	0.0645	0.1275	0.046	0.2105	0.164	0.1395
CCRC-M22	0.3735	0.3995	0.2595	0.402	0.4035	0.366	0.362	0.391	0.242	0.4175	0.3935	0.401
JIM132	0.022	0.0195	0.01	0.038	0.0235	0.0185	0.0105	0.023	0.009	0.048	0.0385	0.0295
JIM1	0.0105	0.026	0.007	0.023	0.033	0.0205	0.009	0.026	0.01	0.043	0.022	0.0265
CCRC-M15	0.0595	0.0825	0.0285	0.072	0.038	0.0645	0.0425	0.036	0.0385	0.081	0.073	0.069
CCRC-M8	0.1325	0.1995	0.0645	0.2335	0.094	0.194	0.1345	0.0805	0.168	0.2635	0.206	0.167
JIM16	0.0485	0.0475	0.0105	0.079	0.056	0.0585	0.0405	0.045	0.031	0.125	0.1	0.0895
JIM93	0.012	0.0285	0.005	0.061	0.0445	0.038	0.025	0.0295	0.017	0.14	0.088	0.0605
JIM94	0.0095	0.028	0.0055	0.0595	0.056	0.0365	0.0255	0.042	0.0145	0.125	0.0695	0.0675
JIM11	0.1235	0.2115	0.0945	0.263	0.274	0.2095	0.182	0.2515	0.125	0.395	0.379	0.3315
MAC204	0.03	0.0465	0.0205	0.164	0.0975	0.0935	0.069	0.0745	0.037	0.181	0.208	0.17
JIM20	0.124	0.188	0.0745	0.2545	0.188	0.164	0.1375	0.1795	0.12	0.339	0.2925	0.2845

JIM14	0.107	0.1705	0.049	0.2305	0.1425	0.115	0.0875	0.1255	0.06	0.268	0.2685	0.247
JIM19	0.2575	0.344	0.2055	0.513	0.391	0.3285	0.284	0.336	0.226	0.595	0.5095	0.6015
JIM12	0.085	0.1255	0.059	0.187	0.154	0.129	0.111	0.128	0.085	0.3285	0.313	0.2355
CCRC-M133	0.1175	0.1185	0.08	0.1125	0.112	0.106	0.171	0.144	0.102	0.1265	0.167	0.152
CCRC-M107	0.146	0.1585	0.0845	0.1295	0.1695	0.1255	0.1875	0.218	0.126	0.1665	0.1935	0.185
JIM4	0.0025	0.0065	0	0.002	0	0.0055	0.002	0	0.0045	0.005	0.005	0.004
CCRC-M31	0.005	0.003	0.0045	0.0015	0.0025	0	0.002	0.006	0.0055	0	0.002	0
JIM17	0.0365	0.085	0.00875	0.0965	0.126	0.0875	0.056	0.099	0.0435	0.1495	0.1255	0.139
CCRC-M26	0.0035	0.006	0.00425	0.0115	0.0025	0.003	0.0025	0.0025	0.003	0.021	0.0145	0.009
JIM15	0.007	0.0165	0.00025	0.011	0.006	0.007	0.0025	0.012	0.0045	0.0155	0.0105	0.0115
JIM8	0.009	0.018	0.0075	0.016	0.011	0.003	0.007	0.0175	0.0065	0.018	0.012	0.0155
CCRC-M85	0.009	0.018	0.01	0.021	0.0115	0.008	0.0045	0.015	0.027	0.0235	0.0185	0.0125
CCRC-M81	0.0115	0.019	0.0075	0.0185	0.0065	0.0075	0.0085	0.0095	0.016	0.016	0.0145	0.0125
MAC266	0.0023	0.0085	0.006	0.0055	0	0	0.004	0.0005	0.00425	0.00025	0.002	0.005
PN16.4B4	0.005	0.007	0.006	0.006	0.0015	0.0045	0.006	0.0005	0.004	0.008	0.0055	0.009
MAC207	0.235	0.2565	0.178	0.386	0.3265	0.274	0.236	0.2605	0.1845	0.421	0.3815	0.3515
JIM133	0.0875	0.106	0.078	0.162	0.106	0.123	0.102	0.1245	0.0885	0.147	0.1635	0.1145
JIM13	0.052	0.088	0.0225	0.103	0.0545	0.0585	0.031	0.051	0.0375	0.123	0.1	0.087
CCRC-M92	0.0925	0.103	0.0765	0.1875	0.1675	0.13	0.108	0.1175	0.0965	0.2455	0.2385	0.2225
CCRC-M91	0.0495	0.0685	0.0385	0.142	0.096	0.093	0.0655	0.0755	0.046	0.2025	0.174	0.171
CCRC-M78	0.135	0.142	0.107	0.2705	0.223	0.1845	0.158	0.1995	0.123	0.286	0.286	0.2825
MAC265	0.002	0.0045	0.0025	0.0045	0.00175	0.001	0.00325	0.0045	0.003	0.00175	0.00225	0.0015
CCRC-M97	0.0095	0.021	0.0075	0.0175	0.0105	0.008	0.0055	0.0155	0.0095	0.022	0.018	0.0165
